# The epidemiology and clinical features of melioidosis in the Lao PDR: a 21-year prospective hospital-based cohort study

**DOI:** 10.1186/s12879-025-11729-1

**Published:** 2025-12-29

**Authors:** The Lao Melioidosis Research Group, Amphayvanh Seubsanith, Amphone Sengduangphachanh, Anisone Chanthongthip, Anousone Douangnouvong, Bounthaphany Bounxouei, Bountoy Sibounheuang, Cathrin E. Moore, Danoy Chommanam, Davanh Sengdatka, David A. B. Dance, Joy Silisouk, Khamla Choumlivong, Khamloun Choumlivong, Khuanta Duangmala, Ko Chang, Koukeo Phommasone, Latsaniphone Boutthasavong, Malavanh Vongsouvath, Malee Seephonelee, Manivanh Vongsouvath, Manivone Simmalavong, Manophab Luangraj, Mayfong Mayxay, Olay Rattana, Onanong Sengvilaipaseuth, Paul N. Newton, Phonepasith Panyanouvong, Punam Mangtani, Sayaphet Rattanavong, Siho Sisouphone, Simmaly Phongmany, Somsavanh Sihalath, Valy Keoluangkhot, Viengmon Davong, Vilada Chansamouth, Xaipasong Xaiyaphet

**Affiliations:** Lao-Oxford-Mahosot Hospital-Wellcome Trust Research Unit, Mahosot Hospital, Vientiane, Lao People’s Democratic Republic

**Keywords:** *Burkholderia pseudomallei*, Melioidosis, Lao PDR, Mahosot Hospital, Epidemiology

## Abstract

**Background:**

Melioidosis, caused by *Burkholderia pseudomallei*, poses a significant health risk in tropical and subtropical regions. The disease is endemic in Southeast Asia, including the Lao PDR (Laos), where the first case was confirmed in 1999. Although diagnostic improvements have led to increased identification of patients, comprehensive clinical and epidemiological data on melioidosis in Laos remain limited.

**Methods:**

We conducted a 21-year prospective study (1999–2020) at Mahosot Hospital and other primary-tertiary hospitals in Vientiane Capital and surrounding provinces. Data were collected for all culture-confirmed melioidosis patients, including demographics, clinical presentations, laboratory findings, and outcomes. The epidemiological and clinical features of melioidosis patients were identified and described.

**Results:**

A total of 1744 patients were culture-positive for *B. pseudomallei*. The majority (77.9%) were adults (aged ≥ 15 years), with a median age of 50 years (IQR 40–60) for adults, and 6 years ,(IQR 4–9) for children (aged < 15 years). Seventy-four percent of infections occurred during the rainy season. Diabetes, including new admission hyperglycaemia, was a major associated co-factor, affecting 48.7% of patients. Chronic comorbidities were present in 46.7% of the cohort and were associated with disseminated disease and higher case-fatality rates (CFR). The overall CFR was 28.9% (34.9% among adults and 9.2% among children) and was higher in older patients, those with disseminated disease, and those with chronic multimorbidity. Patients with a shorter duration of illness before admission had higher CFRs, the highest CFR (42.4%) was seen in those admitted within 7 days of symptom onset, consistent with greater disease severity on hospital arrival.

**Conclusions:**

Melioidosis is endemic in Lao PDR, with high infection and case fatality rates, particularly among vulnerable individuals. Early detection, improved diagnostics and public health interventions are essential to reduce incidence and improve disease outcomes, especially for those with chronic conditions like diabetes. Expanding diagnostic facilities and raising awareness among healthcare workers in remote areas for both melioidosis and diabetes are crucial steps forward.

**Supplementary Information:**

The online version contains supplementary material available at 10.1186/s12879-025-11729-1.

## Background

Melioidosis, caused by the environmental bacterium *Burkholderia pseudomallei*, poses a significant health risk in tropical and subtropical regions. First recognised in Yangon (then Rangoon), Myanmar (then Burma), in 1911 [1], melioidosis is now known to be endemic throughout Southeast Asia, including Thailand, Vietnam, Cambodia, Malaysia and Singapore, and northern Australia [2–11]. There is also growing evidence of a wider global distribution [12].

The Lao PDR (Laos) confirmed its first patient with melioidosis in 1999 at the Microbiology Laboratory of Mahosot Hospital in the capital, Vientiane [13, 14]. This discovery revealed a hidden burden of melioidosis, highlighting gaps in diagnostic capacity and treatment. Improvements in diagnostic techniques and increased awareness among healthcare workers led to a rise in reported cases [15, 16]. Increased presentations of patients with melioidosis during May to October each year is significantly related to the climate, specifically the monsoon season [17].

The Lao-Oxford-Mahosot Hospital-Wellcome Trust Research Unit (LOMWRU), embedded within the Microbiology Laboratory of Mahosot Hospital, Vientiane Capital, established the Unknown Infection (UI) study in 2000 to investigate causes of fever in Laos [16]. As part of this project, we collected clinical data on all confirmed melioidosis patients identified through microbiological cultures.

Awareness of melioidosis and availability of diagnostic facilities remain limited in Laos, mainly due a shortage of skilled laboratory technicians, and essential laboratory equipment. *B. pseudomallei* requires specific culture techniques for optimal isolation, and identification can be a problem for those who are not familiar with its characteristics. Currently, diagnostic capacity is centralised in Vientiane Capital, primarily at Mahosot Hospital and the National Centre for Laboratory and Epidemiology (NCLE) [14].

Although a few reports on melioidosis in Laos exist, a comprehensive description of the clinical and epidemiological characteristics of Lao patients with melioidosis has not yet been published [14, 16, 17]. The study aims to clarify the burden and clinical and epidemiological characteristics of hospitalised melioidosis from 1999 to 2020 in Laos, emphasising its significance as a public health concern and informing health policy decisions to reduce incidence and mortality.

## Methods

### Study design and population

This study included 1744 consenting patients, comprising 1711 enrolled in the UI study [16] and 33 melioidosis patients from the Febrile Illness Evaluation in a Broad Range of Endemicities (FIEBRE) study [18] from 1 October 1999 to 31 December 2020. They comprise both inpatients and outpatients at Mahosot Hospital and other primary to tertiary hospitals in Vientiane, including Mittaphab Hospital, Setthathirath Hospital, 103 Military Hospital, Police Hospital, Children’s Hospital, and Mother and Child Hospital, as well as provincial hospitals. We included patients with a positive culture of *B. pseudomallei* from any biological specimens, such as blood, pus, urine, throat swab, sputum, pleural fluid, or joint aspirate and other body fluids. *B. pseudomallei* was cultured and identified by the Microbiology Laboratory of Mahosot Hospital. Venous blood samples and other relevant specimens from patients suspected of infection were sent for confirmation of melioidosis by culture, following the protocol described by Phetsouvanh et al*.* [16] and by the standard operating procedures (SOPs) for identification of Gram negative bacilli of the Microbiology Laboratory of Mahosot Hospital (detailed in the next section). All culture-positive consenting patients, regardless of age or sex, from 1999 to 2020 were included without exclusion criteria. Only the first episode of culture-positive melioidosis was included in our analysis, although some patients did re-present with clinical or microbiological recurrence during the study period.

### Laboratory procedures

All clinical specimens received by the Microbiology Laboratory underwent bacterial culture. Blood samples were inoculated into paired culture bottles and incubated at 35–37 °C for up to 7 days. The blood culture bottles were examined daily for turbidity, and if turbidity was observed, aliquots from one or both bottles were sub-cultured onto non-selective media (blood agar [Oxoid], incubated in 5–10% CO₂ for 48 h, plus MacConkey agar incubated aerobically and chocolate agars incubated in CO_2_ if Gram negative rods were seen on a Gram stain. “Blind” sub-cultures were also performed onto blood, chocolate and MacConkey agars was performed on day 1 and onto chocolate agar on day 7 following inoculations. Pus and urine specimens, and a centrifuged urine deposits (from 2014 onwards), were cultured directly onto blood agar and Ashdown’s agar and incubated aerobically for up to 4 days. Other specimen types were also cultured on Ashdown’s agar and in selective broth containing colistin (SBCT, made in-house), in addition to other standard media if clinical suspicion of melioidosis existed or Gram-negative bacilli suggestive of *Burkholderia pseudomallei* were observed microscopically. SBCT broth was incubated aerobically, examined daily for 4 days, and sub-cultured onto Ashdown’s agar if turbidity or a surface pellicle was observed. Colonies with typical morphology suggestive of *B. pseudomallei* underwent Gram staining and oxidase testing. Oxidase-positive, Gram-negative bacilli were further confirmed using *B. pseudomallei*-specific latex agglutination testing (Faculty of Tropical Medicine, Mahidol University, Thailand). Isolates from blood and other specimen cultures were additionally confirmed using the API® 20NE biochemical identification system (bioMérieux, Marcy L’Etoile, France). Although isolates were not confirmed in real time using molecular methods such as PCR or sequencing, or Matrix-Assisted Laser Desorption/Ionization Time-of-Flight mass spectrometry, subsequent studies of numerous isolates for other purposes has shown that the presumptive methods used for clinical purposes were highly accurate.

### Data collection

Data were extracted from patient medical records and manually entered using a study ID only into a standard case report form (CRF), then entered into Microsoft Excel. Identifiable information, such as names and addresses, were excluded, with the exception of district and province of residence. Collected data included patient demographics, clinical presentations, laboratory findings from the Laboratory Information Management System (LIMS), and information at discharge.

### Definition of characteristics

Demographic information and clinical presentations were recorded, including seasonality, illness duration before admission, chronic comorbidities, systemic organ involvement, and discharge outcomes. Age was stratified based on the recognised Lao national hospital system. Paediatric patients are less than 15 years and would usually be the responsibility of paediatric wards. In contrast, any patients who are 15 and above would be cared for the adult wards. Occupations were classified as high or low risk: high-risk occupations involved outdoor activities with frequent exposure to soil and water, whereas low-risk occupations involved indoor jobs with minimal exposure (Supplementary Table 1). Seasonality was defined by admission months: the rainy season (May to October) and the dry season (November to April) [19]. Acute melioidosis was classified as symptoms persisting less than 56 days before admission, and chronic melioidosis as symptoms lasting 56 days or more [9]. *B. pseudomallei* infection was categorised as localised or disseminated. Localised melioidosis was defined by a confirmed single focus of infection, excluding bacteraemia, while disseminated melioidosis involved multiple sites or organs, or isolated bacteraemia, or bacteraemia and other foci, including pneumonia [20]. Individual organ involvement, such as lung infections, skin and soft tissue (SST) infections, head and neck (H&N) infections, intra-abdominal abscesses, bone and joint infections, were also noted. We defined patients as having risk factors if they had underlying chronic comorbidities documented in the hospital chart or patient-held health record, consistent with those described in the previous Australian and Thai studies [9, 21]. These included prior diagnoses of diabetes mellitus (DM), chronic kidney disease (CKD), chronic lung disease, chronic liver disease, non-HIV-related immunosuppression, and blood disorders. Diabetes was defined by a documented medical history or elevated blood glucose (hyperglycaemia) on admission. Patients without a prior diagnosis of diabetes but presenting with elevated glucose were also classified as diabetic; however, we acknowledge that some patients may have had stress-induced hyperglycaemia. Due to a lack of follow-up data, we could not definitively confirm diabetes in these individuals.

The study tracked discharge outcomes, distinguishing between survivors and those who were either discharged in a moribund state (discharged at the request of the patient or their family, with family awareness of impending death) or died in-hospital.

### Statistical analysis

Statistical analyses were performed using R version 4.4.1 [22] to identify patterns in the distribution of positive *B. pseudomallei* cultures and describe the epidemiological and clinical features of the patients. Patients’ home villages were mapped to display the geographic distribution of cases and time trends were plotted to illustrate the dynamics of melioidosis cases over time. Categorical variables were summarised as frequencies and percentages, while continuous variables were presented as median with interquartile ranges (IQR) or mean with standard deviations (SD), as appropriate. Associations between categorical variables were assessed using the Chi-square or Fisher’s exact test, depending on the expected frequencies within the contingency tables. For associations between continuous and categorical variables, the Student’s t-test was used or the Wilcoxon rank-sum test if data were non-normal distributed. Due to incomplete documentation in patients’ hospital charts, missing data affected several variables, resulting in differing denominators across analyses.

### Ethical approval

This research adhered to the provisions of the Declaration of Helsinki. Written informed consent was obtained from participants or their legally acceptable representative (LAR) or guardians. Ethical approval for the UI study, in which this study was embedded, was initially granted by the Ethical Review Committee of the Faculty of Medical Sciences, National University of Laos. The approval has been regularly updated, with subsequent approval granted by the Research Ethics Committee, University of Health Sciences, Vientiane, Lao PDR. Additional approval was obtained from the Oxford Tropical Research Ethics Committee (OxTREC), University of Oxford, Oxford, UK; and from the London School of Hygiene and Tropical Medicine Ethics Committee (reference no 29465/RR/32597).

## Results

### Characteristics and age groups stratification

**Fig. 1:**
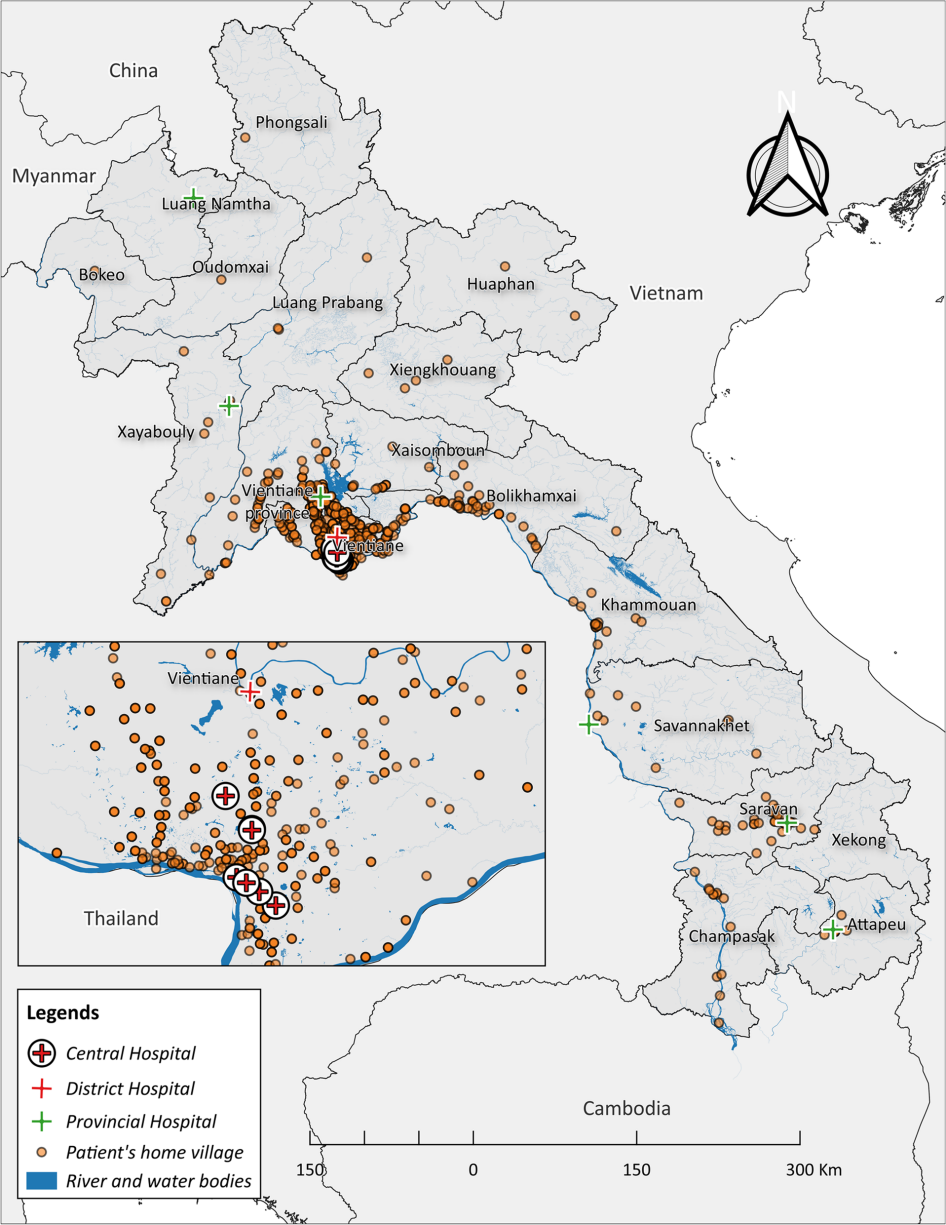
Location of recorded home addresses of melioidosis patients in Laos, 1999–2020 (home village data available for 1694 out of 1744 patients). Note that the only provincial hospitals shown are those to which melioidosis patients were admitted

**Fig. 2:**
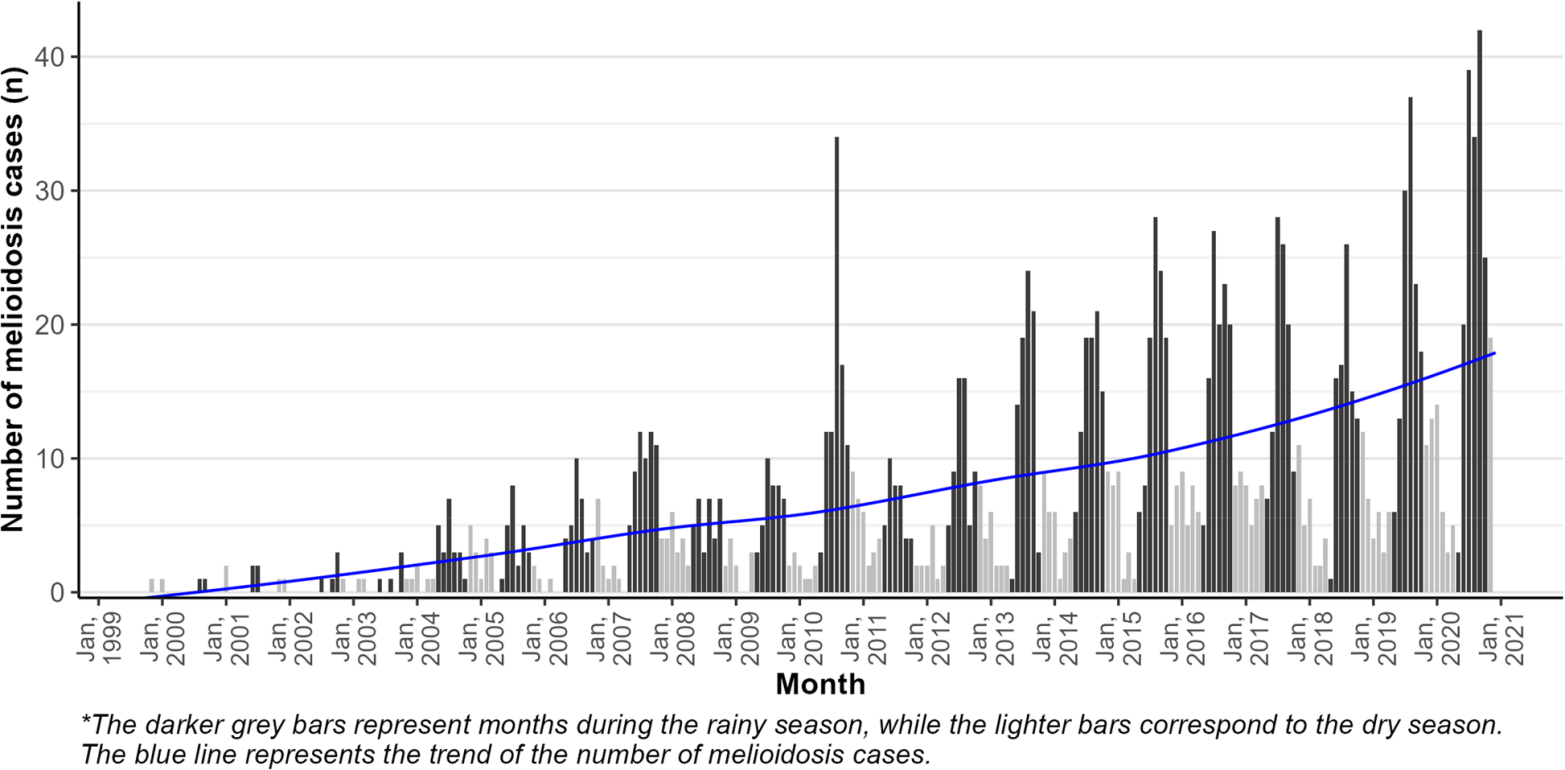
Yearly and monthly time trends in the number of melioidosis patients diagnosed from 1999 to 2020 (*N* = 1744)

Over a 21-year period (255 months), 1744 patients were confirmed to be culture-positive for *B. pseudomallei* in the Microbiology Laboratory of Mahosot Hospital. Of these, 124 patients (7%) were outpatients. During the study period, 12 patients were identified as having recurrent positive specimen culture—ten were adults—, ranging within 1—4 years after discharge (after intensive treatment phase). The cohort included patients from all provinces of Laos except Luang Namtha. The largest number of cases was observed in the central regions, primarily Vientiane Capital, Vientiane, Xaisomboun (new province separated from Vientiane Province since 2013), and Bolikhamxai Provinces, which were also the main sources of clinical samples. Smaller clusters were also identified in the southern part of Laos (Fig. 1). The overall median age was 45 years (IQR 19–57); adults (aged 15 and above, *N* = 1359) had a median age of 50 years (IQR 40–60), while children (less than 15 years, *N* = 385) had a median age of 6 years (IQR 4–9). Occupation was recorded for only 893 adults, with 53.4% classified as having high-risk occupations, and 34% in low-risk roles. Unemployed adults accounted for 12.5%, including retired individuals, jobless persons, students, and monks. Children (aged less than 15 years) were categorised as unemployed. Annual case numbers increased over time, with a surge to more than 100 cases per year after 2010 (Seasonal Mann–Kendall test *P <* 0.001). Most patients (73.7%, 1285/1744) presented during the six-month rainy season (May to October) (Fig. 2) (Supplementary Fig. 2).

The median duration of illness, from onset of symptomatic infection to admission, was 14 days (IQR 7—30 days), with minimal difference between adults and children (*P =* 0.897). Only ten percent of the cohort (140/1452) had infection symptoms persisting for more than 56 days (chronic melioidosis), with no significant difference between children and adults (8.5% vs 10%, *P =* 0.217). Fifty eight percent of the cohort (1012/1742) presented with disseminated disease, which was more common in adults (68.5%, 930/1357) compared to children (21.3%, 82/385, *P <* 0.001) (Table 1). Among 1649 patients with data available (91 patients without blood samples taken, and 5 missing), 891 (54%) were bacteraemic. Bacteraemia was more frequent in adults (63.8%, 832/1305) compared to children (15.3%, 59/385, *P <* 0.001). Of the 891 bacteraemic cases, 669 (75.1%) had identifiable foci of infection outside of the blood stream. Respiratory infections were the most common clinical manifestation, affecting 44.3% of patients, followed by SST and H&N infections, each occurring in approximately 26% of patients. The distribution of specific clinical manifestations related to solid organ infections varied significantly between age groups. Among adults, deep-seated infections were more common than in children, particularly for lung infections (52.7% vs. 15%), SST infections (27.8% vs. 18.5%), intra-abdominal abscesses (14.5% vs. 3.3%), urinary infections (12.7% vs. 0%), and bone and joint infections (7.9% vs. 1.2%) (all *P <* 0.001). In contrast, H&N infections and throat swab (TS) culture-positive cases without a known infection focus were more frequent in children (66.9% and 5.5%, respectively) compared to adults (13.3% and 2.7%, respectively) (*P <* 0.001 and *P =* 0.013, respectively). There was a single case of central nervous system (CNS) involvement identified in an adult, presenting as a brain abscess visible on a CT scan, though surgical drainage and pus culture were not conducted.

**Table 1:** Baseline characteristics of melioidosis patients, stratified by age group (*N* = 1,744)

**Characteristic**	**Overall, n/N (%)**^a^	**Age groups**		***P***^b^
		**Less than 15 years, n/N (%)**^a^	**15 years and above, n/N (%)**^a^	
**Age (year), median (IQR)**	45 (19—57) ^*N*=1744^	6 (4—9) ^*N*=385^	50 (40—60) ^*N*=1359^	< 0.001
**Sex**				0.007
*Female*	724/1744 (41.5)	183/385 (47.5)	541/1359 (39.8)	
*Male*	1020/1744 (58.5)	202/385 (52.5)	818/1359 (60.2)	
**Occupation**				< 0.001
*Low-risk*	304/1278 (23.8)	0/385 (0)	304/893 (34)	
*High-risk*	477/1278 (37.3)	0/385 (0)	477/893 (53.4)	
*Unemployed*	497/1278 (38.9)	385/385 (100)	112/893 (12.5)	
**Season**				< 0.001
*Dry*	459/1744 (26.3)	67/385 (17.4)	392/1359 (28.8)	
*Rainy*	1285/1744 (73.7)	318/385 (82.6)	967/1359 (71.2)	
**Duration of illness (day), median (IQR)**	14 (7—30) ^*N*=1452^	14 (7—28) ^*N*=318^	14 (7—30) ^*N*=1134^	0.897
**Duration of illness (day)**				0.217
*0–7*	482/1452 (33.2)	99/318 (31.1)	383/1134 (33.8)	
*8–14*	362/1452 (24.9)	95/318 (29.9)	267/1134 (23.5)	
*15–28*	199/1452 (13.7)	43/318 (13.5)	156/1134 (13.8)	
*29–42*	255/1452 (17.6)	53/318 (16.7)	202/1134 (17.8)	
*43–56*	14/1452 (1)	1/318 (0.3)	13/1134 (1.1)	
> *56*	140/1452 (9.6)	27/318 (8.5)	113/1134 (10)	
**Disease dissemination**				< 0.001
*Localised*	730/1742 (41.9)	303/385 (78.7)	427/1357 (31.5)	
*Disseminated*	1012/1742 (58.1)	82/385 (21.3)	930/1357 (68.5)	
**Bacteraemia and foci**^c^				< 0.001
*Non-bacteraemic*	758/1649 (46)	285/344 (82.8)	473/1305 (36.2)	
*Bacteraemic without foci*	222/1649 (13.5)	12/344 (3.5)	210/1305 (16.1)	
*Bacteraemic with known foci*	669/1649 (40.6)	47/344 (13.7)	622/1305 (47.7)	
**Respiratory infection**	676/1527 (44.3)	51/341 (15)	625/1186 (52.7)	< 0.001
**SST infection**	383/1494 (25.6)	63/341 (18.5)	320/1153 (27.8)	< 0.001
**H&N infection**	381/1494 (25.5)	228/341 (66.9)	153/1153 (13.3)	< 0.001
**Intra-abdominal abscess**	168/1418 (11.8)	11/338 (3.3)	157/1080 (14.5)	< 0.001
**Urinary infection**	148/1509 (9.8)	0/341 (0)	148/1168 (12.7)	< 0.001
**Bone & joint infection**	94/1485 (6.3)	4/341 (1.2)	90/1144 (7.9)	< 0.001
**CNS infection**	1/1485 (0.1)	0/341 (0)	1/1144 (0.1)	NA
**TS culture-positive only**	51/1513 (3.4)	19/347 (5.5)	32/1166 (2.7)	0.013
**Number of comorbidities**				< 0.001
*No comorbidity*	929/1744 (53.3)	376/385 (97.7)	553/1359 (40.7)	
*Single comorbidity*	621/1744 (35.6)	9/385 (2.3)	612/1359 (45.0)	
*Multimorbidity*	194/1744 (11.1)	0/385 (0)	194/1359 (14.3)	
**Diabetes**				< 0.001
*Non-diabetic and not hyperglycaemic*	751/1464 (51.3)	334/335 (99.7)	417/1129 (36.9)	
*Prior known diabetic*	613/1464 (41.9)	1/335 (0.3)	612/1129 (54.2)	
*Hyperglycaemic without known diabetes*	100/1464 (6.8)	0/335 (0)	100/1129 (8.9)	
**Chronic kidney disease**	149/1447 (10.3)	0/335 (0)	149/1112 (13.4)	< 0.001
**Chronic lung disease**	26/1457 (1.8)	0/335 (0)	26/1122 (2.3)	0.005
**Chronic liver disease**	23/1451 (1.6)	0/335 (0)	23/1116 (2.1)	0.008
**Non-HIV immunosuppressed**	127/1457 (8.7)	2/335 (0.6)	125/1122 (11.1)	< 0.001
**Haematology disease**	32/1460 (2.2)	6/335 (1.8)	26/1125 (2.3)	0.568
**Haematology disease types**				NA
*Anaemia (Unknown cause)*	3/32 (9.4)	0/6 (0)	3/26 (11.5)	
*Aplastic anaemia*	1/32 (3.1)	0/6 (0)	1/26 (3.8)	
*G6PD Deficiency*	1/32 (3.1)	0/6 (0)	1/26 (3.8)	
*Thalassemia*	27/32 (84.4)	6/6 (100)	21/26 (80.8)	
**Discharge status**^d^				< 0.001
*Alive*	1055/1484 (71.1)	315/347 (90.8)	740/1137 (65.1)	
*Died*	429/1484 (28.9)	32/347 (9.2)	397/1137 (34.9)	

*Abbreviation*: *SST* Skin and soft tissue, *H&N* Head and neck, *CNS* Central nervous system, *TS* Throat swab, *P =* *p*-value

^a^Percentages are within columns. Denominators vary due to missing data

^b^Wilcoxon rank sum test; Pearson’s Chi-squared test; NA; Fisher’s exact test

^c^Ninety-one patients did not have blood taken, and missing for four

^d^The “Died” category included patients discharged moribund

Regarding comorbidities, 46.7% of the cohort (815/1744) had chronic underlying conditions. The proportion was dominantly higher in adults (59.3%, 806/1359) compared to children (2.3%, 9/385) (*P <* 0.001). Diabetes, as determined from the patient's history or high admission blood glucose, affected 48.7% of the cohort (713/1464, including 100 patients with hyperglycaemia upon admission without a prior diagnosis of diabetes). Almost all diabetic patients were adults (712/713). Only a single child was diagnosed with type 1 diabetes. Other comorbidities, including chronic kidney, lung and liver diseases, as well as non-HIV immunosuppressive conditions, were observed exclusively in adults. The overall case-fatality rate (CFR) was 28.9% (428/1484), including 44 patients discharged moribund. CFR was higher in adults (34.9%, 397/1137) compared to children (9.2%, 32/347) (*P <* 0.001) (Table 1).

#### Association of organ involvement in bacteraemic versus non-bacteraemic patients

Deep-seated infections, such as those involving the lungs, intra-abdominal abscesses (liver and/or splenic abscesses), urinary tract, and bones and joints, were strongly associated with bacteraemia (all *P <* 0.001). In contrast, superficial infections, including H&N infections (such as parotid abscess and cervical lymphadenitis), SST infections, and solely culture-positive throat swabs (fever without other evidence of infection), were more common in non-bacteraemic patients (*P <* 0.001 for all comparisons) (Table 2).

**Table 2:** Pattern of systemic organ involvement by presence or absence of bacteraemia

**Organ system**	**N**^a^	**With organ involvement, n (%)**^b^	**Non-bacteraemic, n (Row%)**^c^	**Bacteraemic, n (Row%)**^c^	***P***^d^
Respiratory infection	1527	676 (44.3)	163 (24.2)	510 (75.8)	< 0.001
SST infection	1494	383 (25.6)	230 (62.7)	137 (37.3)	< 0.001
H&N infection	1494	381 (25.5)	301 (88.8)	38 (11.2)	< 0.001
Intra-abdominal abscess	1418	168 (11.8)	56 (33.3)	112 (66.7)	< 0.001
Urinary infection	1509	148 (9.8)	35 (23.8)	112 (76.2)	< 0.001
Bone & joint infection	1485	94 (6.3)	13 (13.8)	81 (86.2)	< 0.001
CNS infection	1485	1 (0.1)	0 (0)	1 (100)	NA
TS culture-positive only	1513	51 (3.4)	48 (100)	0 (0)	NA

*Abbreviation*: *SST* Skin and soft tissue, *H&N* Head and neck, *CNS* Central nervous system, *TS* Throat swab, *P =* *p*-value

^a^Ns vary due to incomplete data for some characteristics

^b^Percentages are within columns. Denominators (Column Ns) vary due to incomplete data for some characteristics; analyses used available cases

^c^Percentages are within rows. Denominators (Row Ns) vary due to incomplete data for some characteristics; analyses used available cases

^d^Pearson’s Chi-squared test; NA; Fisher’s exact test

### Association with disseminated disease

Out of 1742 patients (excluding 2 patients with uncertain disease dissemination), patients with disseminated disease (*N* = 1012) had a higher median age of 51 years (IQR 40–60) compared to 22 years (IQR 8–47) for those with localised disease (*N* = 730) (*P <* 0.001) (Supplementary Table 7). The proportion of patients with disseminated disease increased with age and was more common in males (60.8%) than in females (54.2%) (*P <* 0.001, and *P =* 0.006, respectively). Disseminated disease was more frequent among patients with high-risk occupations (73%, *P <* 0.001). A higher proportion of disseminated cases presented during the dry season (61.9%), compared to the rainy season (56.7%) (*P =* 0.053). The median duration of illness before admission was longer among patients with localised disease (18 days, IQR 10–30), compared to those with disseminated disease (10 days, IQR 6–21; *P <* 0.001). Notably, among patients presenting within seven days of symptom onset, the majority had disseminated (72.3%), rather than localised disease (27.7%, *P <* 0.001).

In terms of infection foci, deep-seated infections primarily presented as disseminated disease, including respiratory tract infections (88.9%), intra-abdominal abscesses (91.7%), urinary infections (89.2%), and bone and joint infections (93.6%). In contrast, superficial infections such as SST infections, and H&N infections were more common in those who presented with localised disease (all *P <* 0.001) (Supplementary Table 7). Chronic comorbidities were more common in patients with disseminated disease. Among patients with diabetes (including those with hyperglycaemia on admission), 80.1% presented with the disseminated form (*P <* 0.001). Similarly, a majority of patients with other chronic comorbidities presented with disseminated disease: chronic kidney disease (89.2%), chronic lung disease (88.5%), chronic liver disease (87%), and non-HIV immunosuppression patients (84.3%) (*P <* 0.001, *P =* 0.001, *P =* 0.004, and *P <* 0.001, respectively). When considering the number of comorbidities, disseminated disease was more frequent in patients with at least one co-morbidity compared to those without (*P <* 0.001) (Supplementary Table 7).

### Association with mortality

**Fig. 3:**
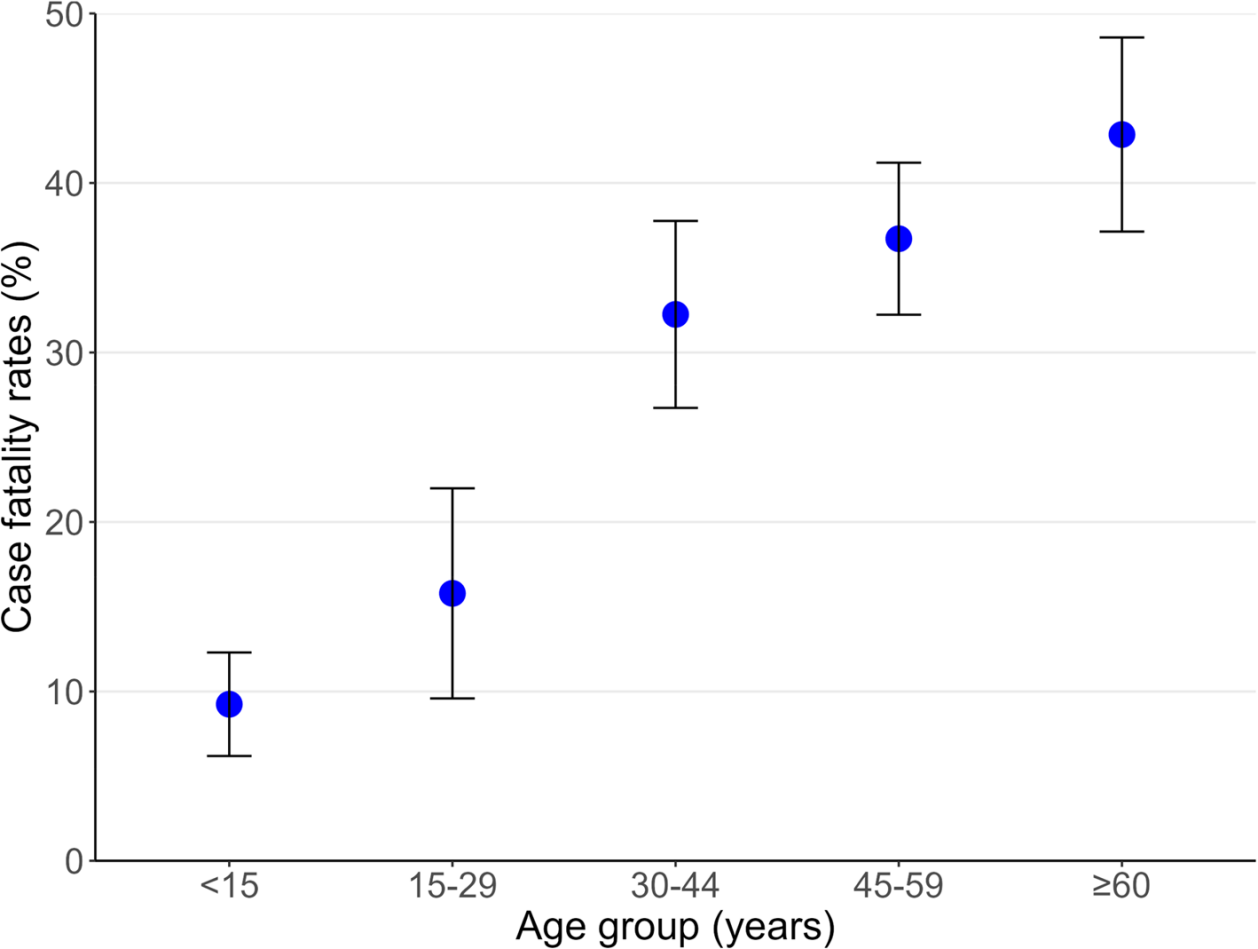
Case-fatality rates by age group, with blue dots representing death percentages and black lines representing 95% confidence intervals

Among 1485 melioidosis patients with outcome data, the case-fatality rate (CFR) was 28.9% (429/1485); differences by demographic and clinical characteristics are summarised in Table 3. Thirty per cent of deaths occurred within 24 h of admission, and most other deaths occurred between 2 and 7 days thereafter (Supplementary Fig. 4). The median age of patients who died and were discharged moribund was higher at 50 years (IQR 40—60), compared to 39 years (IQR 11—54) for survivors (*P <* 0.001). CFRs rose with age, peaking at 43.4% in patients aged 60 and above (*P <* 0.001) (Fig. 3).

**Table 3:** Associations of characteristics with mortality (*N* = 1484)

**Characteristic**	**N**^a^	**Discharge status**		***P***^c^
		**Alive, n/N (Row%)**	**Died, n/N (Row%)**^b^	
**Age (year)**	1484	39 (11—54) ^*N*=1055^	50 (40—60) ^*N*=429^	< 0.001
**Age group**	1484			< 0.001
< *15*		315/347 (90.8)	32/347 (9.2)	
*15–29*		112/133 (84.2)	21/133 (15.8)	
*30–44*		186/275 (67.6)	89/275 (32.4)	
*45–59*		280/443 (63.2)	163/443 (36.8)	
≥ *60*		162/286 (56.6)	124/286 (43.4)	
**Sex**	1485			0.545
*Female*		441/613 (71.9)	172/613 (28.1)	
*Male*		614/871 (70.4)	257/871 (29.6)	
**Occupation**	1198			< 0.001
*Low-risk*		208/297 (70)	89/297 (30)	
*High-risk*		316/457 (69.1)	141/457 (30.9)	
*Unemployed*		380/444 (85.6)	64/444 (14.4)	
**Season**	1484			0.357
*Dry*		273/394 (69.3)	121/394 (30.7)	
*Rainy*		782/1090 (71.7)	308/1090 (28.3)	
**Duration of illness (day)**	1372	14 (7—30) ^*N*=982^	8 (5—16) ^*N*=390^	< 0.001
**Duration of illness (day)**	1372			
*0–7*		258/448 (57.6)	190/448 (42.4)	
*8–14*		250/343 (72.9)	93/343 (27.1)	
*15–28*		146/189 (77.2)	43/189 (22.8)	
*29–42*		194/242 (80.2)	48/242 (19.8)	
*43–56*		13/14 (92.9)	1/14 (7.1)	
> *56*		121/136 (89)	15/136 (11)	
**Disease dissemination**	1483			< 0.001
*Localised*		619/636 (97.3)	17/636 (2.7)	
*Disseminated*		435/847 (51.4)	412/847 (48.6)	
**Blood involvement**^d^	1423			< 0.001
*Non-bacteraemic*		650/678 (95.9)	28/678 (4.1)	
*Bacteraemic*		345/745 (46.3)	400/745 (53.7)	
**Respiratory infection**	1419	330/604 (54.6)	274/604 (45.4)	< 0.001
**SST infection**	1411	310/369 (84)	59/369 (16)	< 0.001
**H&N infection**	1412	360/379 (95)	19/379 (5)	< 0.001
**Intra-abdominal abscess**	1344	117/157 (74.5)	40/157 (25.5)	0.811
**Urinary infection**	1408	54/108 (50)	54/108 (50)	< 0.001
**Bone & joint infection**	1400	61/87 (70.1)	26/87 (29.9)	0.542
**CNS infection**	1402	1/1 (100)	0/1 (0)	> 0.999
**TS culture-positive only**	1409	27/27 (100)	0/27 (0)	0.001
**Diabetic/hyperglycaemic**	1385	411/664 (61.9)	253/664 (38.1)	< 0.001
**Chronic kidney disease**	1372	66/139 (47.5)	73/139 (52.5)	< 0.001
**Chronic lung disease**	1382	13/25 (52)	12/25 (48)	0.019
**Chronic liver disease**	1376	13/20 (65)	7/20 (35)	0.433
**Non-HIV immunosuppressed**	1379	64/113 (56.6)	49/113 (43.4)	< 0.001
**Haematology disease**	1383	29/32 (90.6)	3/32 (9.4)	0.020
**Number of comorbidities**	1485			< 0.001
*No comorbidity*		571/724 (78.9)	153/724 (21.1)	
*Single comorbidity*		391/582 (67.2)	191/582 (32.8)	
*Multimorbidity*		93/179 (52)	86/179 (48)	

*Abbreviation*: *SST* Skin and soft tissue, *H&N* Head and neck, *CNS* Central nervous system, *TS* Throat swab, *P * *p*-value

^a^Percentages are within rows. Denominators (Row Ns) vary due to incomplete data for some characteristics; analyses used available cases

^b^Patients discharged moribund are included in the “Died” category

^c^Wilcoxon rank sum test; Pearson’s Chi-squared test; NA; Fisher’s exact test

^d^Sixty patients did not have blood taken, and missing for two

CFRs did not differ between high- and low-risk occupations (31% vs 30%); comparisons were not possible for unemployed patients because soil–water exposure was uncertain in this group. Similarly, there was also no difference of CFR between the season of presentation (*P =* 0.357).

Patients who died or were discharged moribund had a shorter median duration of illness before admission (8 days, IQR 5—16) than survivors (14 days, IQR 7—30; *P <* 0.001). CFR was highest in those presenting within ≤ 7 days of symptom onset (42.4%) and declined with longer durations (*P <* 0.001). The same pattern was observed in disseminated disease; no duration-related difference was detected in localised disease (Supplementary Table 4a—4c).

Higher CFRs were recorded in patients with bacteraemia (53.7%), lung infection (45.4%) and urinary infection (50%), whereas lower CFRs occurred in superficial infections, such as SST infection (16%), and H&N infection (5%). CFR did not differ between patients with or without intra-abdominal abscesses (*P =* 0.811), or bone and joint infections (*P =* 0.542).

With respect to underlying chronic comorbidities, diabetic or hyperglycaemic patients had a higher CFR than non-diabetic or euglycaemic patients (38.1% (253/664) vs 17.6% (127/721), *P <* 0.001). CFRs were also elevated in patients with CKD (52.5%), chronic lung diseases (48%), and non-HIV immunosuppression (43.4%) compared to those without these comorbidities (*P <* 0.001, *P =* 0.019, and *P <* 0.001, respectively). By contrast, patients with blood disorders had a lower CFR than those without (9.4% vs 28%, *P =* 0.020). Overall, CFR was 32.8% in patients with a single comorbidity, and higher, 48%, in those with multiple comorbidities, compared to 21.1% in patients without comorbidity (*P <* 0.001) (Table 3).

## Discussion

Our 21-year study provides the first detailed clinical and epidemiological description of melioidosis cases in Laos. We confirm endemic *B. pseudomallei* throughout the country except Luang Namtha Province, where the absence of cases may reflect lower environmental contamination or sparse sampling [23–25]. Annual case numbers increased throughout the study. UI-study blood-culture data show a stable total number of blood-culture sets collected, whilst the proportion positive for *B. pseudomallei* continued to rise, supporting our finding (Supplementary Fig. 1). This pattern suggests two possibilities: one, an initial decade in which the increase reflects greater clinical awareness among healthcare workers in both central and rural areas, leading to more specimen’s submission for culture and two, after 2010, a probable true increase in community-acquired melioidosis. Most patients lived near Vientiane Capital, the Mahosot Hospital catchment, but patients’ home villages represent only where they were living at the time of diagnosis, not where they were infected. Some patients from northern Laos may have travelled to other endemic areas elsewhere, including central or southern provinces, or even north-eastern Thailand prior to falling ill, potentially leading to unrecognised infection from those locations. Seventy-four percent of cases occurred in the rainy season, consistent with increased exposure to soil- and water-contact during agriculture. *B. pseudomallei* is widely present in the environment in Laos, especially in wet soil or paddy fields [23–26]. Our findings align with studies from elsewhere in Asia and Northern Australia, where occupational exposure is a major risk in Southeast Asia but only a minor factor in Australia [9, 27–33]. Categorisation of patients by occupation risk in Laos may well be misleading as significant numbers of people in apparently low-risk occupations engage in rice-farming part time, contributing to infection risk [17]. Malaysian findings likewise show higher risk for outdoor workers (e.g., farming, forestry, fishing, and the unemployed) who had a significantly higher chance of contracting severe melioidosis, especially those with chronic underlying diseases [34].

We found differences in disease presentation between adults and children. Adults more often exhibited disseminated disease than children, similar to findings in Thailand [35]. Only one patient had proven central nervous system (CNS) involvement. Other endemic countries, such as Australia and Thailand, report higher rates of CNS involvement [9, 35]. Given Laos’s endemic status and the lack of availability of imaging and neurosurgical services outside Vientiane, the true number is probably higher than identified in the study. Practically, investigation for CNS melioidosis is not routine unless patients exhibit neurological symptoms, and it may be missed without a head CT scan or MRI, which are inaccessible outside Vientiane. Children presented with milder, localised disease, particularly head- and-neck (H&N) infections, mainly parotid abscesses and cervical lymphadenitis, similar to descriptions in Cambodia but unlike the Darwin Prospective Melioidosis Study [10, 33]. Possible reasons for this difference include differential pathogenicity of local *B. pseudomallei* strains in northern Australia and Laos, or more frequent infection by ingestion of contaminated water in Laos. Similar explanations might also account for the relative infrequency of liver abscesses in Australian patients compared to those in southeast Asia [10]. Three-quarters of adult patients had chronic conditions—most commonly diabetes in 54% which was linked to disseminated disease and death, consistent with previous studies [9, 35]. Community diabetes prevalence is ~ 6.2% [36] but rising prevalence will probably increase melioidosis incidence. Other comorbidities (chronic kidney diseases, non-HIV immunosuppression, and thalassemia) were common, as in Thailand and Malaysia [9, 21, 34].

The overall CFR was 29% and was higher in older patients, those with disseminated disease, which commonly involved blood, lungs, urinary tract, and multi-morbidity, consistent with prior studies [21, 34, 37]. The CFR was lower than reported in Thailand (39%) and Cambodia (52%) [21, 38], although it was similar to recent Thai data (25% at one month, and 34% at one year) [35]. However, the true CFR in Laos is likely higher as post-discharge follow up was not possible. Many patients with melioidosis may also remain undiagnosed or misdiagnosed or may die before reaching healthcare services. Adult mortality exceeded children mortality, consistent with previous reports [9, 21, 39]. This likely relates to older individuals being more prone to chronic diseases, leading to more severe manifestations and hence higher mortality. Disseminated disease, especially bacteraemia with deep-seated infection, was strongly associated with death, aligning with findings from elsewhere [9, 21, 37] and the fatal cases had shorter symptoms duration before admission, as in Thai series [37, 40].

During the study, Laos did not have a national guideline for melioidosis treatment; the clinicians therefore followed recommendations in Dance D. Treatment and prophylaxis of melioidosis [20]. Culture-positive melioidosis during the study period was usually treated with ceftazidime or occasionally meropenem, for a minimum of 10 days in the intensive phase, followed by oral eradication therapy with trimethoprim/sulfamethoxazole or co-amoxiclav for at least 12 weeks. Patients were monitored regularly by members of our clinical team and treatment was adapted according to the clinical response, adverse events etc. wherever possible. Unfortunately, it was not always possible to record precise details of each patient’s therapy, particularly during the eradication phase due to difficulties obtaining follow-up information after discharge from hospital. Expensive costs for intensive care and prolonged hospitalisation including antibiotics, coupled with lost income, place substantial strain on families and sometimes results in premature discharge. In our records, only 12 patients were re-admitted due to recurrent positive culture of *B.pseudomallei* (10 adults, 7 of whom were re-admitted within the first two years after discharge) (Supplementary Table 5).

In addition, our study highlights the urgent need for improved diagnostics and public health interventions to manage melioidosis in Laos. Diagnosing and treating melioidosis is particularly challenging in rural areas with limited diagnostic facilities. This gap has likely led to a biased distribution of detected cases in this study, primarily centred around Vientiane Capital, despite melioidosis typically being considered a disease of rural populations. This suggests that many patients in rural areas may go undiagnosed. To mitigate the impact of melioidosis, it is essential to expand access to diagnostic testing across Laos. Equipping healthcare services in rural areas with necessary diagnostic tools and training is likely to expedite treatment and reduce mortality. Microbiology culture is the gold standard, but during most of the study period, culture was available only at Mahosot. Currently it is now being implemented in some provincial hospitals. Rapid colony latex agglutination test has been developed to shorten identification time of *B. pseudomallei*; however the test cannot be used directly with clinical specimens [41]. The Active Melioidosis Detect (AMD), has been developed as a point-of-care test (POCT) to detect *B. pseudomallei* capsular polysaccharide directly from clinical specimens. The test performs well with pus, sputum, blood culture broths and bacterial colonies, but less sensitive in serum, blood and urine samples, although sensitivity improved significantly in severe sepsis cases, suggesting its potential utility in diagnosing severe melioidosis [42]. However, the AMD is not yet commercially available. In addition to microbiological diagnostics, ultrasound and other imaging technologies could help identify liver and splenic abscesses, which are clues to suggest melioidosis [43].

Given that diabetes is the key risk factor for melioidosis, wider screening and blood sugar control may provide cost-effective timely interventions. While awareness of melioidosis among healthcare staff has been high in north-eastern Thailand for many years, public and at-risk population awareness remains low [44, 45]. Similar to campaigns for other national prioritised diseases, public health messages about diabetes and melioidosis should be disseminated via optimal routes, including print and social media and other innovative approaches, particularly before harvesting season, to warn at-risk populations and discuss available interventions [46]. Additionally, educating the general public about diabetes screening and avoiding possibly contaminated soil and water, by promoting the use of protective equipment when exposure is unavoidable, is important [47]. However, addressing barriers such as knowledge, beliefs, and social influences requires a multisectoral approach to ensure the effectiveness of these prevention campaigns [48].

The study has several limitations. First, post-discharge follow-up was not possible, resulting in a possible underestimation of the CFR. Second, the hospital culture-based study design with most of patients being hospitalised may have biased the findings, limiting generalisation of findings to the broader population. Third, it is possible that there was exposure misclassification and incomplete confirmation of comorbidities, especially in patients who deteriorated rapidly or died shortly after admission. Fourth, some clinical data were missing, partly due to incomplete clinical notes, physical examination records and investigation results, resulting in imbalanced case data. Fifth, not all relevant specimens were taken and sent for culture, notably throat swabs and urine from provincial led to us hospitals due to logistic limitations, which possibly misclassifying disease dissemination. Lastly, admission hyperglycaemia without prior history of diabetes could not be distinguished as true diabetes mellitus or stress-induced hyperglycaemia by infection.

## Conclusions

This study underscores that Laos is an endemic country for melioidosis, with high infection and case fatality rates, particularly among older adults and individuals with chronic medical conditions, especially diabetes. Several actions are needed in order to lower the disease burden through improving the management of both melioidosis and diabetes in Laos. Early detection and timely treatment are crucial for these vulnerable populations, hence diagnostic facilities need to be extended beyond Vientiane Capital for both diseases. Awareness among healthcare workers, particularly in remote areas, about the disease and its management, and the importance of optimal management of predisposing conditions such as diabetes, needs to be raised. Expanding public health education about modifying risk factors and reducing exposure to contaminated environments is also essential. Finally, further population-based and clinical research is needed to fully understand the epidemiology of melioidosis in Laos and to develop both more effective prevention, such as vaccination, and treatment strategies.

## Supplementary Information

Supplementary Material 1

## Data Availability

Data can be accessed upon reasonable request from the corresponding author, the director of LOMWRU and the head of the Microbiology Laboratory. Data are securely stored in controlled access facilities at LOMWRU, Microbiology Laboratory, Mahosot Hospital, Vientiane, Lao PDR.
